# Nitrogen-Doped Porous Core-Sheath Graphene Fiber-Shaped Supercapacitors

**DOI:** 10.3390/polym14204300

**Published:** 2022-10-13

**Authors:** Qianlan Ke, Yan Liu, Ruifang Xiang, Yuhui Zhang, Minzhi Du, Zhongxiu Li, Yi Wei, Kun Zhang

**Affiliations:** 1 Key Laboratory of Textile Science & Technology, Ministry of Education, College of Textiles, Donghua University, Shanghai 201620, China; 2 Center for Civil Aviation Composites, Donghua University, Shanghai 201620, China

**Keywords:** nitrogen doping, small-sized graphene core-sheath, graphene fiber, supercapacitors

## Abstract

In this study, a strategy to fabricate nitrogen-doped porous core-sheath graphene fibers with the incorporation of polypyrrole-induced nitrogen doping and graphene oxide for porous architecture in sheath is reported. Polypyrrole/graphene oxide were introduced onto wet-spun graphene oxide fibers by dip-coating. Nitrogen-doped core-sheath graphene-based fibers (NSG@GFs) were obtained with subsequently thermally carbonized polypyrrole/small-sized graphene oxide and graphene oxide fiber slurry (PPY/SGO@GOF). Both nitrogen doping and small-sized graphene sheets can improve the utilization of graphene layers in graphene-based fiber electrode by preventing stacking of the graphene sheets. Enhanced electrochemical performance is achieved due to the introduced pseudo-capacitance and enhanced electrical double-layered capacitance. The specific capacitance (38.3 mF cm^−2^) of NSG@GF is 2.6 times of that of pure graphene fiber. The energy density of NSG@GF reaches 3.40 μWh cm^−2^ after nitrogen doping, which is 2.59 times of that of as-prepared one. Moreover, Nitrogen-doped graphene fiber-based supercapacitor (NSG@GF FSSC) exhibits good conductivity (155 S cm^−1^) and cycle stability (98.2% capacitance retention after 5000 cycles at 0.1 mA cm^−2^).

## 1. Introduction

With the emerging electronic textiles, the demand for rechargeable energy storage technologies is urgently needed [1,2]. Fiber-based flexible supercapacitors have the characteristics of lightweight, good flexibility, high power density, great cycle stability, and excellent charge–discharge performance [3,4,5,6,7]. Various types of fiber-shaped supercapacitors have been widely studied in recent years.

Owing to the remarkable electrical conductivity, higher tensile strength, and controllable structure [8,9,10], graphene fibers are considered as promising electrode materials for flexible fiber-shaped supercapacitors. However, the graphene nanosheets are easily aggregated and often closely packed in graphene fibers which hinder ion and electron transport in electrodes due to strong π–π interaction between graphene interlayers, resulting in limited specific surface area and thus power and energy density [11,12,13,14]. Therefore, it is necessary to prevent the restacking of graphene nanosheets, and to activate the graphene nanosheets for efficient utilization for energy storage [15]. For example, Zhang et al. [16] made atomic-level modification efforts to limit the restacking and improve the accessibility with the electrolytes to facilitate the ion and electron transport. Tian et al. [17] introduced heteroatom groups in the graphene surfaces is one of the most promising methods. Nitrogen atoms share a comparable atomic size with carbon atoms but have higher electronegativity than that of carbon [18], showing promise in n-type doping of carbon materials. The nitrogen doping leads to enhanced electrochemical properties in organic, aqueous solution, and ionic liquid electrolytes, which may open a new avenue for the research on the chemical doping of carbon-based materials and their electrochemical device applications [19]. N doping can enhance the surface polarity and improve surface wettability of graphene-based materials [20,21].The nitrogenous active site can efficiently facilitate charge transfer and enhance the electrochemical activity in graphene-based materials [22,23,24]. One method for preparing nitrogen-doped graphene fibers is by uniformly mixing nitrogen-containing substance with graphene oxide solution for wet spinning with further carbonization. For example, Ding et al. [25] obtained nitrogen-doped graphene fiber by wet spinning blended pyridine and graphene oxide solution. Guan et al. [26] used the microfluidic method to obtain nitrogen-doped graphene fibers by injecting a mixture of graphene oxide and urea solution into microtubes with further thermal carbonization. However, the nitrogen source may destroy the structure of graphene fibers. Currently, there are limited studies to achieve both porous structures and superior electrochemical performances.

In this work, nitrogen-doped graphene fiber electrodes by dip-coating wet-spun graphene oxide fibers with slurry containing small-sized graphene oxide and pyrrole monomers as nitrogen sources and thermal carbonization are designed. The influence of mass ratio of different components on the electrochemical performance of graphene fiber electrodes was systematically investigated. The graphene fibers were successfully doped with nitrogen atoms. Moreover, the nitrogen doping resulted in 159.7% enhancement of area specific capacitance (38.3 mF cm^−2^) and 159.5% enhancement of energy density 3.40 μWh cm^−2^), compared with that of the pure graphene fibers. NSG@GF FSSC also demonstrated ultralong cycling life and good conductivity.

## 2. Materials and Methods

### 2.1. Materials

The graphite oxide powder was bought from the Sixth Element Materials Technology Co., Ltd., Changzhou, China. Polyvinyl alcohol (PVA, 99%) was purchased from Sigma-Aldrich, Shanghai, China. Pyrrole (PY), ferric chloride (FeCl_3_, 98%), and sulfuric acid (H_2_SO_4_, 98 wt.%) were purchased from Chemical Reagent Co., Ltd., Shanghai, China. Deionized (DI) water was made with Master-Q15 (resistivity~18.3 MΩ cm), Shanghai, China.

### 2.2. Preparation of Small-Sized Graphene Oxide (SGO)

A certain amount of graphite oxide was dispersed in abundant DI water and subsequently ultrasonicated by a Biosafer with a power of 312 W for 1 h to form graphene oxide (GO) solution. To remove the unexfoliated graphite oxide, the above dispersion was centrifuged at 3000 rpm for 15 min. Then the supernatant was centrifugated at 8000 rpm for 15 min to remove the small-sized graphene oxide, and condensed to serve as the solution for wet spinning graphene oxide fibers.

The small-sized graphene oxide (SGO) (~150 nm in diameter) was prepared by breaking graphene oxide into smaller pieces with high-power ultrasonication for 20 h. Then the SGO dispersion was centrifuged at 3000 rpm for 30 min to obtain SGO with suitable sizes. Lastly the SGO dispersion was thermally concentrated to a suitable concentration (15 mg mL^−1^) for further wet spinning.

### 2.3. Preparation of GF

The graphene oxide fiber (GOF) was wet spun by the method described in our earlier work [27]. A total of 15 mg mL^−1^ GO dispersion was injected into an ethanol/DI water (1:3 *v*/*v*) coagulation bath containing 5 wt.% CaCl_2_ through a spinneret (inner diameter of 0.3 mm) at room temperature. By mounting onto a syringe pump (LSP02-1B), the continuous wet-spun GOFs were drawn out from the coagulation and washed by DI water and ethanol. The GOFs were then wound onto a winder and dried under tension by infrared drying at 100 °C for 4 h.

### 2.4. Preparation of SG@GFs

Small-sized graphene-based fibers (SG@GFs) were fabricated by the method as described below. First, the as-prepared GOFs were immersed in the SGO solution, and dried and solidified at 80 °C. The resultant fibers are denoted as SGO@GOF. SG@GF was prepared by pre-oxidized SGO@GOFs with multiple temperature steps at 120 °C, 150 °C, and 180 °C for 1 h. Then, they were thermally annealed at 800 °C for 3 h under nitrogen protection to form SG@GFs.

### 2.5. Preparation of NSG@GFs

Core-sheath nitrogen-doped graphene-based fibers (NSG@GFs) were prepared by the following two-step method. The core fiber is GOF, the sheath was fabricated of polypyrrole in SGO. The PPY/SGO@GOFs were fabricated by the “dip-coating” method. First, a certain amount of pyrrole was mixed into SGO slurry. Then, the mixture was homogenized with a high shear dispersion device (FLUKO, FA25-D) at 10,000 rpm for 15 min and cooled in an ice bath for 3 h. Then GOF were dipped in the above mixture for sheath coating, and then immersed in FeCl_3_ solution to polymerize pyrrole monomers in the SGO sheath. Then, PPY/SGO@GOF was finally obtained after drying at 60 °C for 1 h.

NSG@GFs were prepared by pre-oxidizing PPY/SGO@GOFs with multiple temperature steps at 120 °C, 150 °C, and 180 °C for 1 h, respectively. The pre-oxided PPY/SGO@GOFs were thermally annealed by a high-temperature vacuum tubular furnace (OTF-1200X) under nitrogen (N_2_) purge with a heating rate of 2.5 °C/min. Then the core-sheath NSG@GF was produced at 800 °C for 3 h, towards the final required NSG@GFs.

### 2.6. Assembly of NSG@GF-Based FSSCs

The NSG@GF-based FSSCs in aqueous electrolyte were prepared with the following method. A total of 1 g PVA powder was dissolved in 10 mL DI water at 90 °C. The PVA solution was vigorously stirred for 2 h until fully dissolved. Subsequently, 0.98 g H_2_SO_4_ (98 wt.%) was added and stirred at room temperature to prepare PVA/H_2_SO_4_ gel electrolyte. To fabricate quasi-solid-state fiber-shaped supercapacitor, two fiber electrodes with length of ~7 mm were placed in parallel on a flexible polyethylene terephthalate (PET) substrate with an electrode spacing of ~2 mm. Each end of the fibers was connected to polished copper foils by silver paste. Then assembled NSG@GF FSSCs were solidified for 24 h at room temperature to make the electrolyte sufficiently infiltrate into fiber electrodes.

### 2.7. Characterization

Scanning electron microscopy (SEM, HITACHI, TM3000, and SU5000) was used to characterize the morphological feature of GF, SG@GF, and NSG@GF samples. The diameters of GF, SG@GF, and NSG@GF were measured with polarizing optical microscope (NIKON ECLIPSE LV100POL). The microstructure and compositional element distribution of the NSG@GF were further investigated by using transmission electron microscopy (TEM, JEM-2100) coupled with energy dispersive spectrometer (EDS) mapping. Element valence states and contents were preceded on an X-ray photoelectron spectroscopy (XPS, ESCALAB250Xi). Fourier transform infrared spectroscopic (FTIR) measurements were conducted on a Nicolet NEXUS-670; where the resolution is 4 cm^−1^, the scanning wave number range is 4000–400 cm^−1^, to characterize the chemical structure of GOF, GF, SG@GF, and NSG@GF, where KBr was used to mix with samples to prepare thin films for FTIR measurements.

Electrochemical tests were carried out on an electrochemical workstation (CHI 660E, CH Instruments Inc., Bee Cave, TX, USA) with a two-electrode configuration for analysis of cyclic voltammetry (CV), galvanostatic charge/discharge (GCD) and electrochemical impedance spectroscopy (EIS, 0.01 Hz to 100 kHz). The electrical conductivity (σ) was measured with four-wire resistivity measurement method at room temperature.

## 3. Results and Discussion

**Figure 1:**
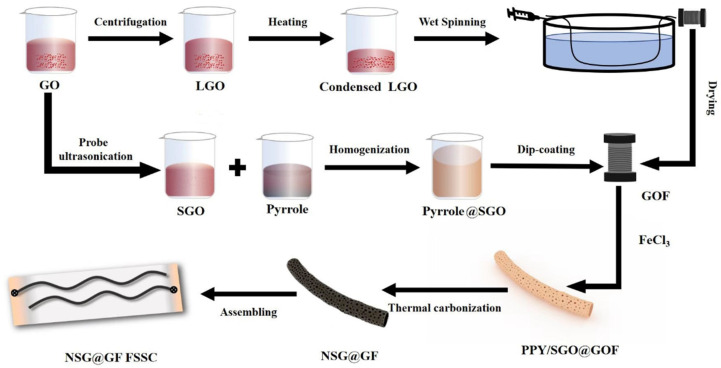
Schematic illustration for the preparation of PPY/SGO@GOF and NSG@GF FSSC.

Figure 1 shows the fabrication process of NSG@GF. The preparation of NSG@GF was performed by combining wet spinning and dip-coating with subsequent thermal carbonization. The core GOF was wet spun by using concentrated LGO dispersion. At the same time, pyrrole monomers were added into aqueous SGOs solution. FeCl_3_ solution was then carefully dropped into the above mixture to produce PPy@SGO slurry. The slurry was then dip-coated onto GOF due to the hydrophilic characteristics between them. Finally, thermal carbonization was conducted to form the designed core-sheath porous graphene-based composite fiber, NSG@GF. For electrochemical measurements, NSG@GF FSSC was assembled by immersing two pieces of NSG@GF into PVA/H_2_SO_4_ gel electrolyte and aligning them in parallel and dried under ambient condition. SG@GF was fabricated by the as-prepared GOF immersed in the SGO solution with subsequent thermal carbonization.

**Figure 2:**
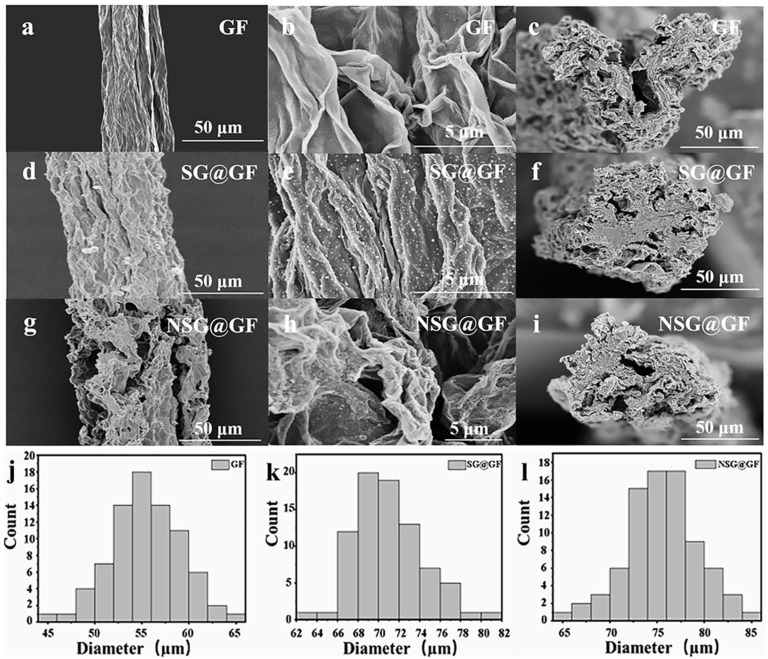
SEM images and diameter of GF, SG@GF, and NSG@GF. (**a**,**b**) Surface image of as-prepared GF. (**c**) Cross-sectional image of as-prepared GF. (**d**,**e**) Surface image of as-prepared SG@GF. (**f**) Cross-sectional image of as-prepared SG@GF. (**g**,**h**) Surface image of as-prepared NSG@GF. (**i**) Cross-sectional image of as-prepared NSG@GF. Diameter distributions of GF (**j**), SG@GF (**k**), and NSG@GF (**l**) obtained by optical microscopy.

Figure 2 shows the SEM images of GF, SG@GF, and NSG@GF. Figure 2a exhibits the surface morphology of GF, showing aligned graphene sheets along the fiber axis, which is attributed to the drawing force during the wet spinning process. The fiber surface shows a typical wrinkled morphology as seen in Figure 2b. Figure 2c shows the cross section of GF, which does appear round, probably due to the irregular shrinkage caused by the rapid evaporation of water.

In comparison, the surface of SG@GF shows less wrinkled morphology along the fiber axis as seen in Figure 2d,e. However, it possesses a slightly round cross-section and apparent interface between core and sheath layers as seen in Figure 2f. Figure 2j shows the distribution of GF diameters with an average diameter of ~55 μm. While, in Figure 2k, SG@GF shows the average diameter of ~72 μm. The average diameter of GF and SG@GF was compared, and it was indicated that SGO solution was successfully coated onto GOF.

It can be clearly seen from Figure 2g,h that nitrogen-doping method involves porous structure [27] on the surface of NSG@GF, which may be able to increase the specific surface area of NSG@GF. In Figure 2i, NSG@GF shows a blurred interface between the core and the sheath, which should be attributed to the existence of C-C cross-link between the graphene layers in both core and sheath [28,29]. The mean diameter distribution of NSG@GF is 75 μm as seen in Figure 2l.

**Figure 3:**
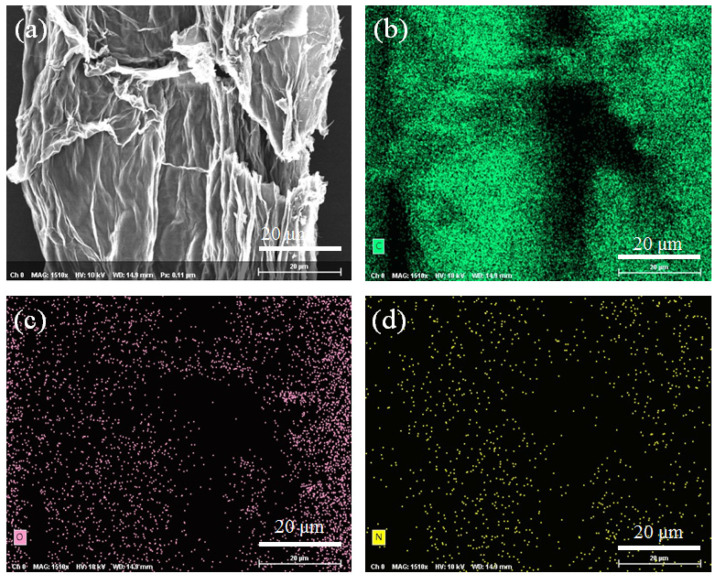
(**a**) EDX images of NSG@GF and (**b**–**d**) element distribution of carbon, oxygen, and nitrogen.

To further identify the nitrogen doping, EDS was conducted to characterize the element distribution of NSG@GF. As seen in Figure 3a–d, carbon (C), nitrogen (N), and oxygen (O) elements are evenly distributed on the NSG@GF, indicating the successful nitrogen doping.

**Figure 4:**
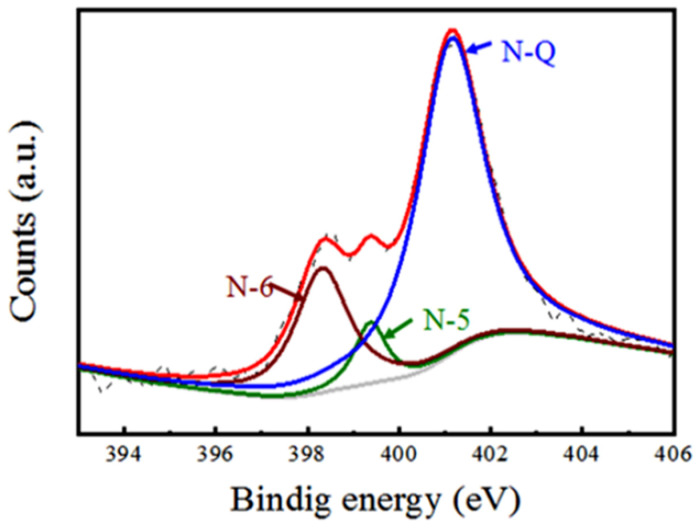
XPS spectra for N1s of NSG@GF.

To quantitively characterize the valency and nitrogen doping level, we conducted XPS characterization for NSG@GF. Figure 4 shows N1s spectrum which can be deconvoluted into three different peaks. There are three types of N species in NSG@GF [30,31], which are pyridinic-N (N-6) [32], pyrrolic-N (N-5) [33], and quaternary N (N-Q) [34] locating at 398.4 eV, 399.4 eV, and 401.1 eV, respectively. Different nitrogen doping configurations have different effects. N-6 is easy to go through redox reaction and provide pseudo-capacitance because of its lower energy band values [33]. N-5 forms a five-membered ring structure [35]. N-5 mainly exists on the edge of the graphene nanosheets which can provide additional electrochemical active sites to enhance pseudo-capacitance [36]. N-Q is doped within the graphitic basal plane, which can improve the electrical conductivity and enhance fast charge/discharge [37]. It can be seen from Figure 4 that the N atoms in NSG@GF mostly exist in the form of N-Q and N-6. Hence, the nitrogen-doping method is believed to show large impact on the improvement of electrochemical performance due to the pseudo-capacitive and highly electrical conductive contributions [38].

**Figure 5:**
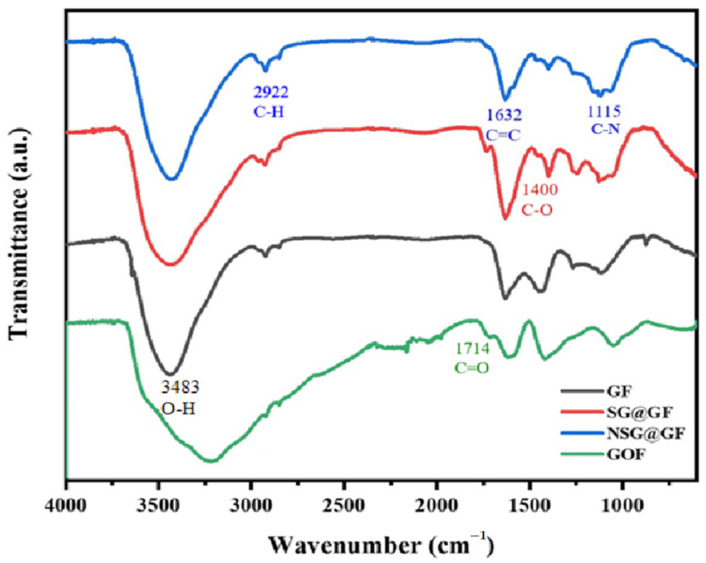
FTIR spectra of GOF, GF, SG@GF, and NSG@GF.

Furthermore, Figure 5 shows the FTIR patterns of the GO, GOF, SG@GF, and NSG@GF. Distinct peaks are visible at 3483 cm^−1^, 1714 cm^−1^, and 1400 cm^−1^, which originate from -OH, C=O, and the C-O bond, respectively. this indicates that the surfaces of GF, GOF, and SG@GF contain abundant oxygen-contained groups [36]. However, the absorption peaks of -OH, C=O, and C-O become smaller or even disappear after thermal carbonization. Moreover, NSG@GF shows additional characteristic peaks at 2922 cm^−1^ (-CH), 1632 cm^−1^ (C=C), and 1115 cm^−1^ (C-N), confirming that NSG@GF is heavily reduced and doped with nitrogen atoms, which has good agreement with XPS results.

**Figure 6:**
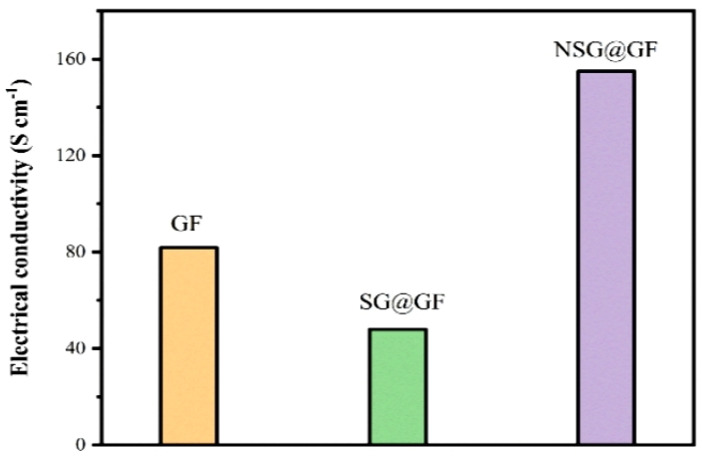
Electrical conductivities of GF, SG@GF, and NSG@GF.

It is seen from Figure 6 that the electrical conductivity of GF, SG@GF, and NSG@GF is 81.79 S cm^−1^, 47.96 S cm^−1^, and 155 S cm^−1^, respectively. The decrease in the conductivity of the SG@GF is due to the increase in the diameter and the internal resistance of the fibers, due to the stacking of the GO layer. In contrast, the increase in the conductivity of the fiber after the nitrogen doping coating is due to the improvement of the electrical conductivity structure of the fiber [39].

**Figure 7:**
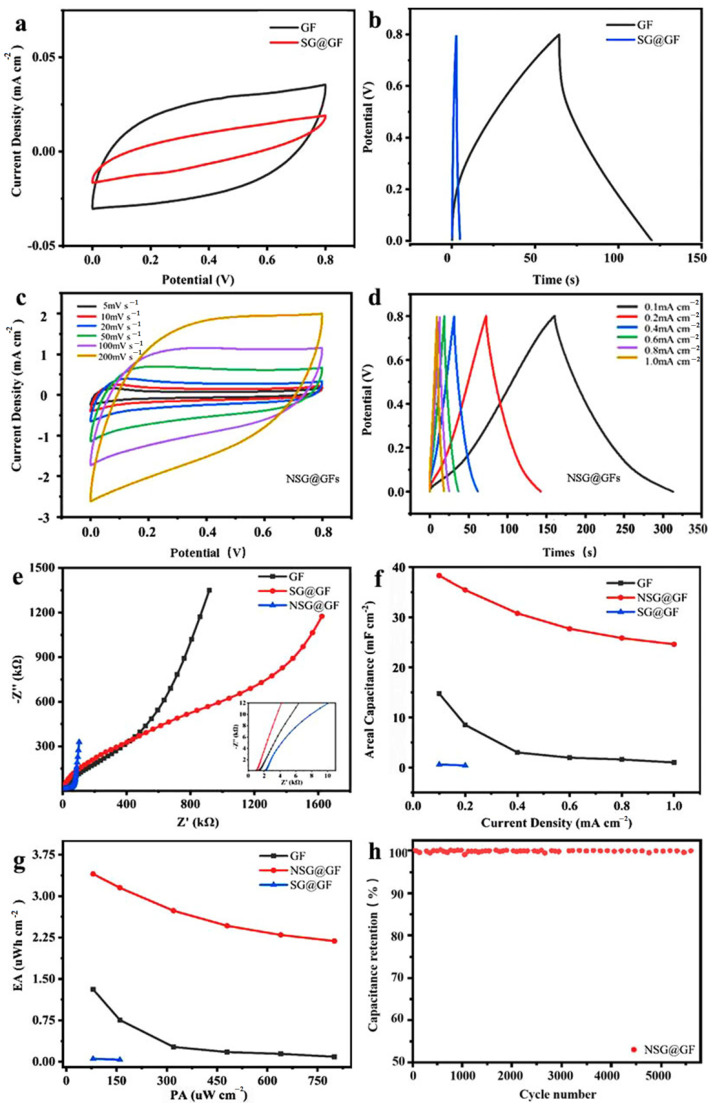
Electrochemical performances of GF, SG@GF, and NSG@GF. (**a**) CV curves of GF, SG@GF measured at a scan rate of 5 mV s^−1^. (**b**) GCD curves of GF, SG@GF measured at a current density of 0.1 mA cm^−2^. (**c**) CV curves of NSG@GFs measured at different scanning rate. (**d**) GCD curves of NSG@GFs measured at different current density. (**e**) Nyquist plots of GF, SG@GF, and NSG@GF. (**f**) Specific capacitances (CA) of GF, SG@GF, and NSG@GF based on GCD test and measured current density. (**g**) The Ragone plots of the FSSCs for GF, SG@GF, and NSG@GF graphene-based fiber electrodes. (**h**) Cycle life of NSG@GF.

The electrochemical properties of GF, SG@GF, and NSG@GF were summarized in Figure 7. Figure 7a shows the CV curves at 5 mVs^−1^ for GF and SG@GF, of which the shapes are nearly rectangular [40], indicating typical double-layer capacitance behavior [41]. The curve area of SG@GF is smaller than that of GF, indicating that pure SG coating deteriorates the electrochemical performance, which may be caused by dense SG coating and poor electrical conductivity.

The GCD curves of GF and SG@GF electrode were measured at a current density of 0.1 mA cm^−2^ (Figure 7b). Compared with that of GF (~320 mV), SG@GF has a large voltage drop (IR drop) of 820 mV, indicating the high internal resistance in SG@GF. Moreover, the discharge time for SG@GF is 2.4 s, which is much smaller than that of pure GF. These results confirm the poor structure of direct coating small graphene sheets on GF. Hence, further modification to the graphene-based composite fiber electrodes is needed for better electrochemical performance.

In Figure 7c, all CV curves of NSG@GFs at different scanning rates demonstrate a rectangular-like shape but with broad peaks, indicating rapid current response to voltage scanning, and suggesting typical electrical double-layer capacitor behavior [3]. However, the broad peak in these curves is considered to be redox reaction during scanning. It is believed to be caused by the nitrogen doping which may have provided extra pseudo-capacitance for enhanced electrochemical performance. The CV curves at 5 mVs^−1^ for NSG@GF show the smallest CV curve area compared with the other scanning rates. More importantly, it is obvious that the CV curve area of NSG@GFs are much larger than those of GF and SG@GF electrodes (Figure 7a), confirming that the introduction of nitrogen doping can significantly enhance the electrochemical performance. The CV curves at 200 mVs^−1^ for NSG@GF shows the largest enclosed area of its CV curve.

Furthermore, the discharging curves (Figure 7d) are not symmetric to its corresponding charge counterpart even at low current density (0.1 mA cm^−2^), indicating the existence of pseudo-capacitance from redox reactions on the shell surface with nitrogen-doped areas in graphene. Compared with the data for GF and SG@GF (Figure 7b), NSG@GF possesses much longer discharging time (~153 s), suggesting much higher energy storage capability.

Figure 7e shows the Nyquist plots of GF, SG@GF, and NSG@GF electrodes. Since electron transfer limited process can be shown by a high frequency, the diffusion process is reflected by a low frequency [42]. In the low frequency region, the NSG@GF has a higher slope, which indicates that the nitrogen-doped fiber has a better charge storage capacity [36]. However, the slope of the pure SG@GF is very small, even lower than that of GF, which indicates that its electrochemical performance is deteriorated [43,44]. Equivalent series resistance (ESR) of GF, SG@GF, and NSG@GF is 1.323 kΩ, 0.9699 kΩ, and 2.076 kΩ. The effect on equivalent series resistance of GF, SG@GF, and NSG@GF is in accordance with the trend in electrical conductivity.

Based on these GCD curves, we calculated their specific capacitances with respect to the current density from 0.1 to 1 mA cm^−2^ (Figure 7f). It shows that the areal specific capacitance of GF at 0.1 mA cm^−2^ is 14.75 mF cm^−2^; however, SG@GF only has a specific capacitance of 0.6 mF cm^−2^. After nitrogen doping, the areal specific capacitance of NSG@GF is significantly improved to 38.3 mF cm^−2^, which is believed to be ascribed to the synergetic effect of nitrogen doping and porous structure. Ragone plots for graphene-based fiber supercapacitor is shown in Figure 7g. The SG@GF had the lowest EA (0.05 μWh cm^−2^), lower than that of GF (1.31 μWh cm^−2^), but the EA of NSG@GF was improved to 3.40 μWh cm^−2^ after nitrogen doping.

As observed in Figure 7h, NSG@GF-based FSSC exhibits good cycling performance with the capacitance retention rate of 98.2% over 5000 cycles, illustrating its superior cyclic stability with a long cycle life.

## 4. Conclusions

In summary, we report a novel strategy to fabricate high-performance FSSCs assembled by nitrogen-doped core-sheath graphene fibers incorporated by slurry containing small-sized graphene oxide and pyrrole monomers serve as nitrogen sources. NSG@GF FSSCs display high electrical conductivity and excellent electrochemical performance. Moreover, the as-assembled FSSCs exhibit good capacitance and cyclic stability. The facile nitrogen-doped method, unique core-sheath graphene-shaped structure, and superior electrochemical properties endow a new avenue in the fields of fiber-shaped energy storage devices.

## Data Availability

The raw data presented in this study are available upon request from the corresponding author.
